# Single-Cell Transcriptomic Characterization of DNCB-Induced Mouse Model Reveals Atopic Dermatitis-Associated Skin Lesions in Skin Microenvironment

**DOI:** 10.1007/s10753-025-02391-5

**Published:** 2026-01-24

**Authors:** Wenxiang Liu, Zhuoya Qiu, Jialei Fu, Lijing Hou, Xiaoyan Ding, Haitao Du, Yanhong Zhai, Zheng Cao, Ping Wang, Cheng Wang

**Affiliations:** 1 Department of Laboratory Medicine, Beijing Obstetrics and Gynecology Hospital, Capital Medical University, Beijing Maternal and Child Health Care Hospital, Beijing, China; 2 Institute of Traditional Chinese Medicine Pharmacology, Shandong Academy of Chinese Medicine, Jinan, Shandong 250014 China; 3 College of pharmacy, Shandong University of Traditional Chinese Medicine, Jinan, Shandong 250014 China

**Keywords:** DNCB, Atopic dermatitis mouse model, Single-cell RNA sequencing, Inflammation, Immune

## Abstract

Atopic dermatitis (AD) is a chronic inflammatory skin disorder characterized by complex cellular heterogeneity. While several studies have begun to characterize the single-cell transcriptomic landscape of human AD, comparable high-resolution data from widely used mouse models remain lacking. Here, we employed single-cell RNA sequencing to profile skin from a 2,4-dinitrochlorobenzene (DNCB)-induced AD mouse model, which recapitulates key pathological features of human AD and is frequently used for mechanistic and therapeutic investigations. In this study, we identified 21 transcriptionally distinct clusters encompassing 11 major cell types. DNCB exposure resulted in robust immune infiltration, including Th2/Th17-skewed T cells, monocytes, macrophages, and inflamed endothelial cells. Keratinocytes were depleted and exhibited hyperproliferation, apoptosis, and differentiation defects, while novel fibroblast subsets displayed impaired maturation and pro-inflammatory states. Intercellular communication analysis revealed monocytes as dominant ligand-receptor signaling hubs. This study provides the first comprehensive single-cell atlas of the DNCB-induced AD model, revealing immune dysregulation, epithelial remodeling, and stromal reprogramming. Notably, the transcriptomic parallels with human AD validate the model’s translational relevance. Our findings establish a foundational reference for future AD research using the DNCB mouse model and offer valuable insights for interpreting drug and target discovery studies performed in this context.

**Supplementary Information:**

The online version contains supplementary material available at 10.1007/s10753-025-02391-5.

## Introduction

Atopic dermatitis (AD), a common chronic inflammatory skin condition, exhibits hallmark features including severe itching, impaired epidermal barrier function, and dysregulated immune responses [1–3]. According to the Global Burden of Disease study, atopic dermatitis affects approximately 15–20% of children and up to 10% of adults worldwide [4], representing a major public health challenge due to its chronic course and substantial impact on quality of life. Although recent advances in biologic therapies (e.g., IL-4Rα antagonists) have achieved breakthroughs [5, 6], some patients with moderate-to-severe exhibit inadequate therapeutic responses, underscoring the urgent need for deeper mechanistic understanding. The skin functions as a complex immune ecosystem, where multiple cell populations engage in dynamic interactions during disease development [7]. While the underlying mechanisms remain incompletely understood, single-cell transcriptomic technologies now offer powerful tools to systematically dissect these cellular dynamics.

The 2,4-dinitrochlorobenzene (DNCB)-induced murine model has become a cornerstone in AD research due to its faithful recapitulation of core disease features, including epidermal hyperplasia, inflammatory microenvironment, and skin barrier dysfunction with increased transepidermal water loss [8–10]. The MC903 (calcipotriol) model has been widely used to study atopic-like inflammation, as it strongly induces epidermal TSLP expression and promotes downstream activation of ILC2s, basophils, mast cells, and dendritic cells, leading to robust Th2-type immune responses [11–14]. However, this model primarily relies on the TSLP-TSLPR axis, raising concerns regarding its translational relevance, since calcipotriol is not an endogenous trigger in human AD pathogenesis and is more commonly associated with irritant contact dermatitis [15, 16]. By contrast, DNCB induces a mixed immune response characterized by an initial Th1/Th17-dominant phase that shifts toward Th2-driven inflammation upon repeated application [17]. This is accompanied by chronic epidermal thickening and pruritus [18], thereby recapitulating the biphasic and relapsing nature of human AD. Moreover, DNCB-induced lesions respond to standard AD therapeutics [19], further supporting their clinical relevance. However, previous investigations have been constrained by their focus on isolated cell populations or signaling pathways, lacking comprehensive characterization of the cutaneous cellular landscape. Key gaps remain in understanding cell-specific transcriptional networks regulating keratinocyte differentiation, myeloid polarization, and vascular remodeling, limiting therapeutic target identification.

Single-cell RNA sequencing (scRNA-seq) represents a transformative solution to these limitations [20]. This cutting-edge technology not only resolves cellular heterogeneity obscured by bulk sequencing but also enables precise delineation of spatiotemporal dynamics in cell-cell communication networks within pathological microenvironments [21, 22]. While several studies have successfully employed scRNA-seq to map human AD lesions [23–25], high-resolution data from animal models remain strikingly scarce [26]. This interspecies data disparity critically impairs the translation of preclinical findings into therapeutic strategies.

In this study, we conducted single-cell transcriptomic analysis of DNCB-induced AD mouse models, characterizing molecular alterations across cutaneous cell populations. Our results confirm the model’s ability to recapitulate key human AD features and identify monocytes as inflammatory signaling hubs. These findings advance our understanding of AD pathogenesis and suggest potential therapeutic targets.

## Materials and Methods

### Animals

Male BALB/c mice at 6 weeks of age were obtained from Jinan Pengyue Experimental Animal Breeding Co., Ltd. (Jinan, China). All experimental protocols complied strictly with the Guide for the Care and Use of Laboratory Animals and received ethical approval from Shandong Academy of Chinese Medicine (Approval No. SDZYY20240304004). Housing was conducted in an SPF facility with regulated environmental parameters, including a 12-hour photoperiod. Standard rodent chow and water were available without restriction.

After a one-week acclimation period, ten mice were randomly assigned to either the control group or the group subjected to DNCB-induced modeling, following a previously established protocol with slight adjustments [10, 27]. One day prior to the experiment, dorsal hair was removed under anesthesia. In the DNCB group, mice received topical application of 100 µL of 1% DNCB (Sigma-Aldrich, St. Louis, MO, USA) dissolved in a vehicle solution (acetone: olive oil = 1:3, v/v) on days 1, 4, and 7 to sensitize the shaved dorsal skin. Subsequently, 100 µL of 0.5% DNCB was subsequently applied to the same area and ears on days 14, 17, 20, 23, 26, 29, and 32 to induce AD-like skin inflammation. Control mice were treated with an equivalent volume of vehicle alone on the same schedule. On day 33, i.e., one day after the completion of the treatment cycle, mice were anesthetized with 2–2.5.5% isoflurane (RWD Life Science Co., Ltd., R510-22-10) in an oxygen mixture and euthanized by cervical dislocation. Dorsal skin tissues were collected for further analysis.

### Skin Tissue Digestion and scRNA-seq

Single-cell suspensions were prepared by pooling skin tissues from five individual mice per group, followed by enzymatic digestion using Collagenase IV and Dispase II at 37 °C for 45–60 minutes under constant agitation. After digestion, samples underwent erythrocyte lysis with ACK buffer and cell viability was assessed using Trypan Blue exclusion. High-quality single-cell suspensions were subsequently processed for barcoding and cDNA library construction using the 10x Genomics Chromium Single Cell 3’ Reagent Kit (v3 chemistry) following the manufacturer’s standardized protocol. Final libraries were subjected to paired-end sequencing (150 bp) on an Illumina NovaSeq 6000 platform to achieve sufficient sequencing depth for robust transcriptome analysis. Following established scRNA-seq protocols, we performed rigorous quality control using Seurat (v4.3.0) and DoubletFinder (v2.0.4) to remove low-quality cells and doublets. Cell clusters were visualized via UMAP dimensionality reduction and annotated based on differentially expressed genes identified by FindAllMarkers function.

### Single-Cell Pseudotemporal Ordering

Pseudotemporal transcriptional dynamics of keratinocytes and fibroblasts were analyzed using Monocle 2 (version 2.26) implemented in R [28]. Cell subsets were extracted from the integrated Seurat object based on cluster annotation and converted into a CellDataSet compatible with Monocle. Size factors and dispersions were estimated, and genes exhibiting high variance across cells were selected for ordering. Dimensionality reduction was carried out using the DDRTree algorithm, and cells were ordered along the pseudotime trajectory to reconstruct potential differentiation or activation paths. Pseudotime heatmaps and gene expression trends were visualized to identify key genes associated with cellular state transitions.

### Differential Expression Analysis of Single-Cell Transcriptomes

Differentially expressed genes (DEGs) between experimental groups were performed using the FindMarkers function in the Seurat package (version 5.1) in R. Cells were annotated according to experimental conditions (e.g., DNCB and CON groups) in the metadata. For each cell cluster or defined cell type, DEGs were identified using the Wilcoxon rank-sum test implemented in the FindMarkers function. DEGs were defined as genes with an adjusted *p*-value < 0.05 and |log₂ fold change| > 0.25. Prior to analysis, raw counts were normalized and scaled according to Seurat’s standard workflow. Visualization of DEGs was conducted via volcano plots to highlight significant expression changes between groups.

### Functional Enrichment Analysis of DEGs

The ClusterProfiler package (version 4.6) in R was employed to conduct functional enrichment analysis on the DEGs. Gene Ontology (GO) enrichment analysis was conducted for biological process categories. KEGG pathway enrichment analysis was conducted to identify significantly overrepresented pathways, with adjusted *p*-values < 0.05 considered statistically significant. Results were visualized with dot plots and enrichment maps to illustrate key biological functions and pathways associated with DEGs.

### Cell-Cell Communication Analysis

CellChat (v1.6) in R was employed to examine cell-to-cell communication networks [22]. Normalized single-cell gene expression data along with cell type annotations were used as input. The identifyOverExpressedGenes and identifyOverExpressedInteractions functions were applied to infer ligand–receptor pairs. Communication probability was computed based on known ligand–receptor databases, and signaling pathways were analyzed to reveal interaction networks among cell populations. Results were visualized through heatmaps, circle plots, and chord diagrams to illustrate intercellular signaling patterns.

### Immunofluorescence Staining for MKI67

Paraffin-embedded skin Sect. (5 μm) were first deparaffinized and rehydrated, then subjected to antigen retrieval in sodium citrate buffer. Sections were blocked in 5% BSA and incubated overnight at 4 °C with anti-MKI67 antibody (ab15580, Abcam). The following day, sections were treated with a fluorophore-labeled secondary antibody (A0516, Beyotime) and counterstained with DAPI. Images were acquired using a confocal microscope. MKI67-positive cells were counted along the basal layer of the epidermis in multiple non-overlapping segments, and the average number per 200 μm width was used for statistical analysis.

### Immunohistochemistry

Skin sections embedded in paraffin were deparaffinized and rehydrated, followed by antigen retrieval in citrate buffer using microwave heating. Endogenous peroxidase activity was quenched with 3% hydrogen peroxide for 10 min, followed by blocking with 5% bovine serum albumin for 30 min at room temperature. Primary antibody incubation was performed overnight at 4 °C on the sections using the following antibodies: anti-CD3 (17617-1, Proteintech), anti-S100A9 (26992-1, Proteintech), anti-CD206 (DF4149, Affinity Biosciences), and anti-CD31 (28083-1, Proteintech). Following washes, sections were treated with HRP-conjugated secondary antibodies (A0258, Beyotime) and stained using a DAB (ZLI-9017, ZSGB-BIO, China) chromogenic substrate. Hematoxylin was used for counterstaining. Images were acquired with a bright-field microscope. The positive cells were counted along the basal layer of the epidermis in multiple non-overlapping segments, and the average number per 200 μm segment used for analysis.

### TUNEL Staining

Apoptotic cells were identified using the TUNEL assay kit (A113-02, Vazyme, Nanjing, China), according to the manufacturer’s instructions. Briefly, paraffin-embedded sections were deparaffinized, rehydrated, and treated with proteinase K for permeabilization. After incubation with the TUNEL reaction mixture at 37 °C for 1 h in the dark, followed by nuclear counterstaining with DAPI. Fluorescence images were captured using a microscope. Quantification of TUNEL-positive cells was performed along the basal layer of the epidermis by counting cells in multiple discrete, non-overlapping regions. The average number of apoptotic cells per 200 μm segment was calculated for statistical comparison.

### Multiplex Cytokine Analysis

Levels of multiple cytokines and chemokines in mouse serum were measured using the Bio-Plex Pro™ Mouse Chemokine Panel 31-Plex (12009159, Bio-Rad) according to the manufacturer’s instructions. Samples were incubated with antibody-coated magnetic beads and analyzed using the Bio-Plex 200 system (Bio-Rad). Concentrations of analytes were determined based on standard curves generated by Bio-Plex Manager software.

### Statistical Analysis

Data analysis was conducted using GraphPad Prism 8 or R. Results are shown as mean ± standard deviation (SD). The unpaired two-tailed Student’s *t*-test was used to compare differences between two groups. Significance thresholds were set at *p* < 0.05 and *p* < 0.01 for statistical and high significance, respectively. For bioinformatics analyses, R software (v4.2.1) was used for data visualization and statistical analysis following standard protocols.

## Results

### scRNA-seq Uncovers Altered Cellular Heterogeneity in the Skin of DNCB-Induced AD Mouse Models

**Fig. 1:**
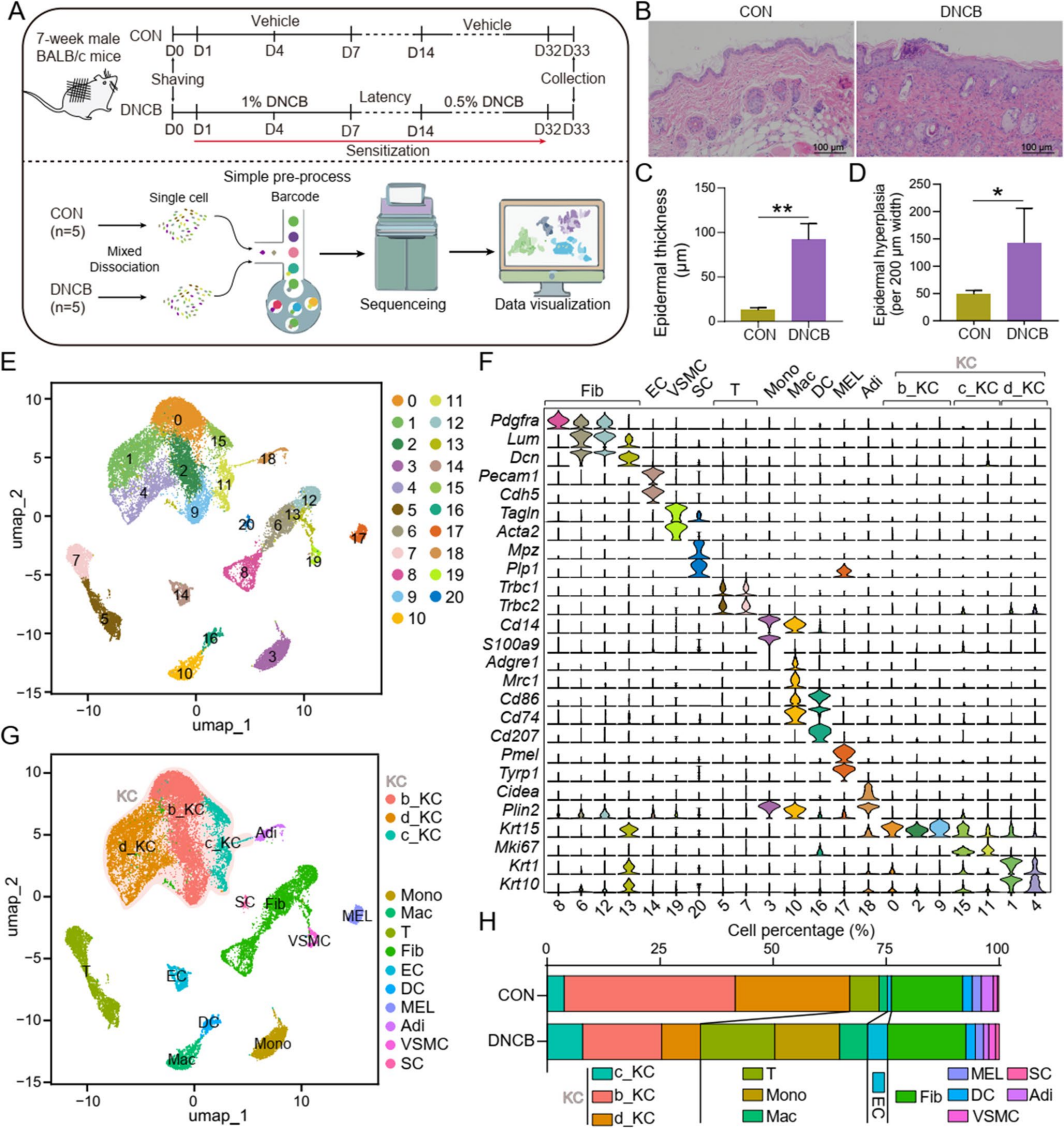
Single-cell transcriptome profiling of skin samples from control and DNCB-induced atopic dermatitis (AD) mice. (**A**) Schematic illustration of the DNCB-induced atopic dermatitis model and sample preparation workflow for scRNA-seq of control and DNCB-treated mouse skin. (**B**) Mouse dorsal skin lesions of control and DNCB-treated groups. (**C **and **D**) The bar plot shows the comparison of epidermal thickness and epidermal hyperplasia between the control and DNCB-treated groups. (**E**) Uniform manifold approximation and projection (UMAP) projection of single-cell profiles reveals 21 cell clusters. (**F**) Violin plots of expression distribution for exemplary cluster-specific marker genes. (**G**) UMAP projection of single-cell profiles reveals 11 cell clusters. (**H**) The percentages of cell types from different groups. Fib: Fibroblasts; EC: Endothelial cells; VSMC: Vascular smooth muscle cells; SC: Schwann cells; T: T lymphocytes; Mono: monocytes; Mac: Macrophages; DC: Dendritic cells; MEL: Melanocytes; Adi, Adipocytes; b_KC: Basal keratinocytes; c_KC: Cycling keratinocytes; d_KC: Differentiating keratinocytes. *: *P* < 0.05; **: *P* < 0.01

The DNCB-induced mouse model is widely recognized as a canonical experimental system for recapitulating AD-like pathology and investigating disease mechanisms and therapeutic interventions [10, 27]. However, the transcriptional landscape of DNCB-induced AD skin remains poorly characterized. In this study, we employed the standard induction protocol (Fig. 1A, top panel) to successfully establish AD-like phenotypes, including characteristic epidermal hyperplasia (Fig. 1B and D). As outlined in the schematic (Fig. 1A, bottom panel), skin samples from each experimental group (*n* = 5 per group) were pooled prior to enzymatic dissociation into single-cell suspensions for scRNA-seq library preparation. Following quality control to exclude low-quality cells and doublets, 12,547 and 12,020 high-quality cells were retained from the control and DNCB-treated groups, respectively, for further analysis (Figure S1A).

Integration and unsupervised clustering identified 21 cell clusters, which were classified into 10 distinct cell types according to canonical markers: fibroblasts (Fib: *Pdgfra*^*+*^, *Lum*^*+*^, *Dcn*^*+*^), endothelial cells (EC: *Pecam1*^*+*^, *Cdh5*^*+*^), vascular smooth muscle cells (VSMC: *Tagln*^*+*^, *Acta2*^*+*^), schwann cells (SC: *Mpz*^*+*^, *Plp1*^*+*^), T cells (T: *Trbc1*^*+*^, *Trbc2*^*+*^), monocytes (Mono: *Cd14*^*+*^, *S100a9*^*+*^, *Adgre1*^*−*^), macrophages (Mac: *Cd14*^*+*^, *Adgre1*^*+*^, *Mrc1*^*+*^, *Cd86*^*+*^), dendritic cells (DC: *Cd74*^*+*^, *Cd207*^*+*^), melanocytes (MEL: *Pmel*^*+*^, *Tyrp1*^*+*^), adipocytes (Adi: *Cidea*^*+*^, *Plin2*^*+*^), and keratinocytes (KC: *Krt15*^*+*^, *Mki67*^*+*^, *Krt1*^*+*^, *Krt10*^*+*^) (Fig. 1E and G, and S1B-S1D). Compared to the control group, DNCB-induced AD mouse skin exhibited a significant increase in T cells, monocytes, macrophage, and endothelial cells, accompanied by a marked reduction in keratinocytes (Fig. 1H). Notably, among the keratinocyte populations, cycling keratinocytes (c_KC) were markedly expanded, whereas basal keratinocytes (b_KC) and differentiating keratinocytes (d_KC) were significantly decreased (Fig. 1H), a pattern that closely resembles the cellular alterations observed in human AD [24].

### DNCB-Induced Multi-Dimensional Keratinocyte Dysregulation: Concurrent hyperproliferation, Enhanced Apoptosis, and Differentiation Defects

**Fig. 2:**
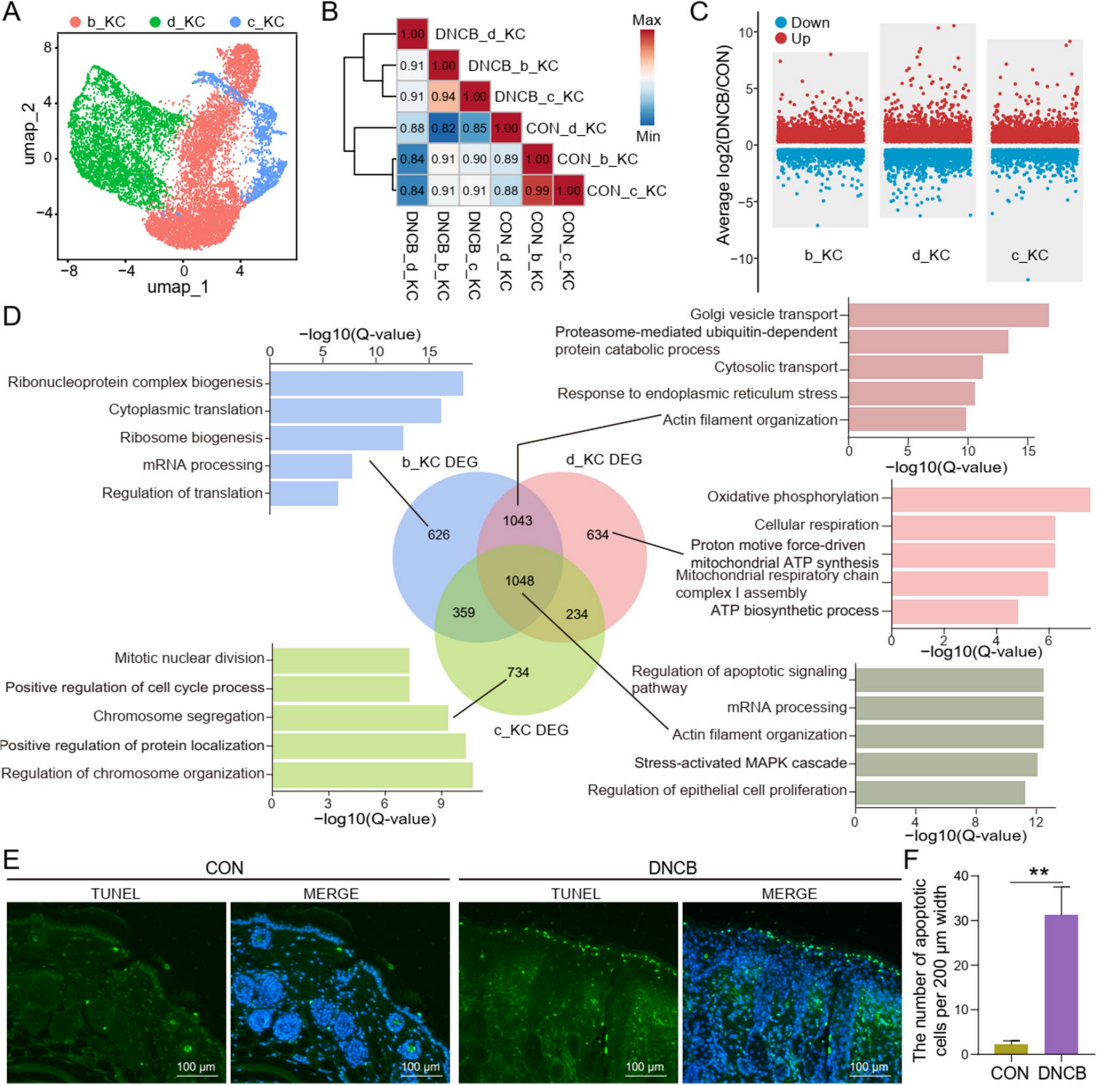
Keratinocyte cell transcriptional profiles are significantly altered in the DNCB-treated group. (**A**) Subpopulation of keratinocyte clusters colored based on three cell subtypes. (**B**) Spearman correlation test was performed by comparing the expression of marker genes among three KC subtypes between the control and DNCB-treated groups. (**C**) Volcano plots of differential analysis between the control and DNCB-treated groups based on three cell subpopulations. (**D**) Venn diagram of DEGs across three keratinocyte subclusters with GO enrichment analysis of biological processes. (**E **and **F**) Representative TUNEL staining images and corresponding statistical bar graphs of skin sections from control and DNCB-treated groups. **: *P* < 0.01

Given that KCs constitute the predominant cell population in the skin, we extracted three KC subtypes for downstream analysis (Fig. 2A). Correlation analysis revealed distinct transcriptional differences among the KC subpopulations, as well as between the treatment and control groups (Fig. 2B). Consequently, we performed differential gene expression analysis separately for each KC subtype to investigate the specific and shared transcriptomic responses to DNCB exposure (Fig. 2C). Functional enrichment analysis of the subgroup-specific DEGs revealed that b_KC cells were predominantly associated with transcriptional processes, including “ribonucleoprotein complex biogenesis”; d_KC cells were enriched in mitochondrial-related pathways, such as “oxidative phosphorylation”; and c_KC cells were primarily linked to proliferative functions, including “regulation of chromosome organization” (Fig. 2D). These findings are consistent with the putative biological characteristics of each keratinocyte subset. Importantly, the shared DEGs across all three subsets were predominantly enriched in apoptosis-related pathways, including the “regulation of apoptotic signaling pathway” (Fig. 2D). Supporting this, TUNEL staining demonstrated a substantial increase in apoptotic keratinocytes in DNCB-treated skin, highlighting apoptosis as a key pathological outcome of allergen exposure (Fig. 2E and F).

**Fig. 3:**
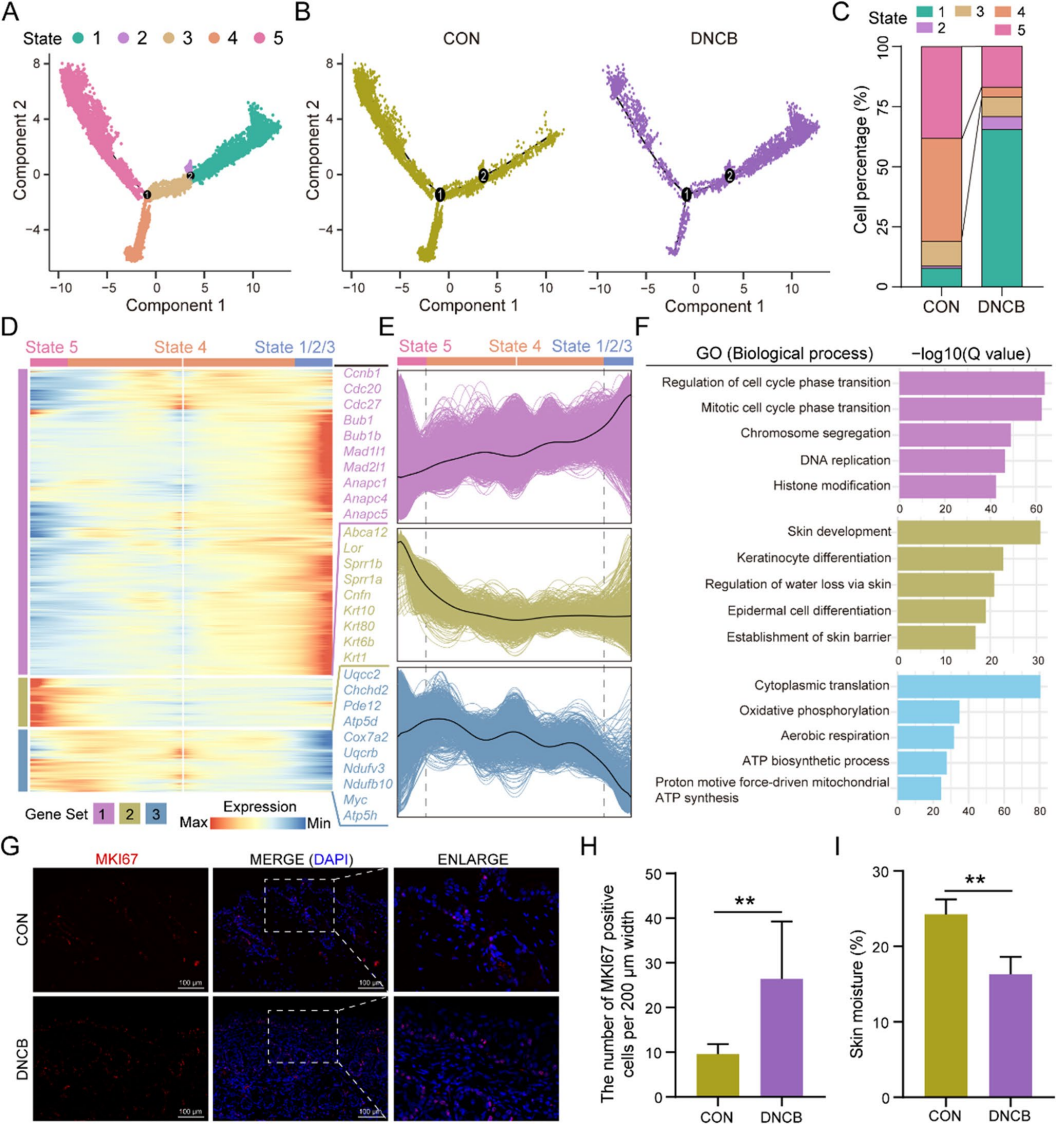
Keratinocyte cell fate is significantly altered in DNCB-treated group. (**A**) Single-cell pseudotime developmental trajectory of keratinocyte populations colored by five states, which were identified with two developmental fates. (**B**) Pseudotime trajectory of keratinocyte from the control and DNCB-treated groups. (**C**) Cell percentages of five states among two sample groups. (**D**) Pseudotime ordered heatmap of three DEG sets between two obvious fates at branch point one. (**E**) The graph shows the expression of the three DEG sets in the pseudotime trajectory. (**F**) The enrichment of GO terms in each gene set. (**G **and **H**) Representative MKI67 staining images and corresponding statistical bar graphs of skin sections from control and DNCB-treated groups. (**I**) The bar plot shows the comparison of skin moisture between the control and DNCB-treated groups. **: *P* < 0.01

Subsequently, we performed pseudotime analysis of keratinocytes using the Monocle2 package, which revealed five distinct cellular states along the differentiation trajectory (Fig. 3A). Interestingly, based on branch point 1, we observed that the combined proportion of cell states 1/2/3 was significantly elevated in the DNCB-treated group, whereas states 4/5 were markedly reduced, compared to the control group. These findings indicate that DNCB exposure profoundly alters the differentiation fate of keratinocytes (Fig. 3B and C). Furthermore, genes exhibiting significant dynamic changes along the pseudotime trajectory were classified into three gene modules (Fig. 3D and E). Functional enrichment analysis revealed that genes highly expressed in states 1/2/3 were primarily involved in cell cycle-related processes, while genes highly expressed in states 4/5 were mainly associated with epidermal barrier integrity and mitochondrial function (Fig. 3F). Consistently, MKI67 immunostaining demonstrated a substantial increase in keratinocyte proliferation upon DNCB treatment (Fig. 3G and H). Concurrently, skin moisture levels significantly decreased (Fig. 3I), suggesting that the hyperproliferative state of keratinocytes was accompanied by excessive water loss from the skin.

### DNCB Induces Activation and Accumulation of Immune Cell Populations in AD Mouse Skin

**Fig. 4:**
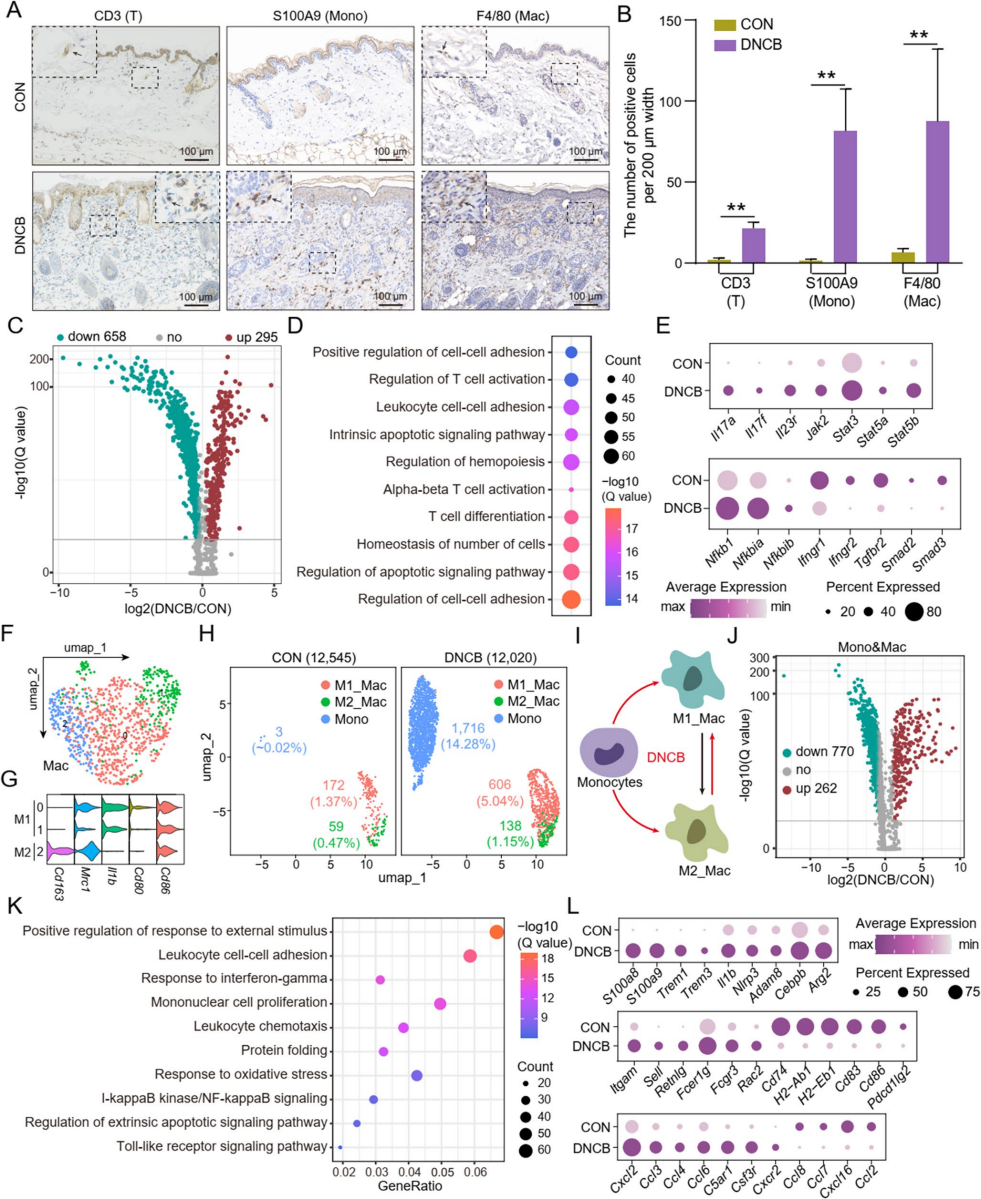
Immune cell populations are significantly altered in DNCB-treated group. (**A **and **B**) Representative IHC images and corresponding statistical bar graphs of CD3, S100A9, and F4/80 between the control and DNCB-treated groups are shown. Scale bar, 200 μm. (**C**) The volcano plot shows the DEGs of T cells between the control and DNCB-treated groups. (**D**) The bubble chart shows the GO (biological process) enrichment results of DEGs in T between the control and DNCB-treated groups. (**E**) Dot plot showing DEGs in T cells from control and DNCB-treated groups. (**F**) Cluster analysis of macrophage with UMAP plots based on cell clusters. (**G**) Violin plots of expression distribution for exemplary M1/M2 macrophage-specific marker genes. (**H**) Extraction of monocytes and M1/M2 macrophage clusters from the UMAP map. (**I**) DNCB treatment induces monocytes and M1/M2 macrophage differentiation. (**J**) The volcano plot shows the DEGs of macrophage cells between the control and DNCB-treated groups. (**K**) The bubble chart shows the GO enrichment results of DEGs in macrophage between the control and DNCB-treated groups. (**L**) Dot plot showing DEGs in macrophage from control and DNCB-treated groups. **: *P* < 0.01

In the DNCB-treated group, CD3⁺ T cells, S100A9⁺ monocytes, and F4/80⁺ macrophages were significantly more abundant than in the control group (Fig. 4A and B), indicating pronounced immune cell infiltration and activation within lesional skin. DEG analysis of T cells showed that significantly downregulated genes outnumbered upregulated ones by more than twofold, with enrichment in pathways related to T cell activation and differentiation (Fig. 4C and D). Notably, key genes associated with Th17 and Th2 polarization, including *Il17a*, *Il17f*, *Il23r*, *Jak2*, *Stat3*, *Stat5a*, and *Stat5b*, were significantly upregulated (Fig. 4E). In addition, pro-inflammatory NF-κB signaling components (*Nfkb1*, *Nfkbia*, *Nfkbib*) were also markedly activated. In contrast, Th1-related interferon-γ pathway genes (*Ifngr1*, *Ifngr2*) and TGF-β signaling molecules (*Tgfbr2*, *Smad2*, *Smad3*) were significantly downregulated (Fig. 4E). These findings suggest that DNCB exposure skews T cell differentiation toward a Th2/Th17-dominant profile.

UMAP analysis of macrophages revealed three transcriptionally distinct subclusters (0/1/2, Fig. 4F). Based on the expression of classical M1 macrophages (M1_Mac) markers (*Cd86*, *Cd80*, *Il1b*) and M2 macrophages (M2_Mac) markers (*Mrc1*, *Cd163*), clusters 0 and 1 were identified as M1_Mac, whereas cluster 2 was classified as M2_Mac (Fig. 4G). UMAP analysis demonstrated that DNCB exposure markedly expanded the populations of Mono (0.02% to 14.28%), M1_Mac (1.37% to 5.04%), and M2_Mac (0.47% to 1.15%) (Fig. 4H). The more pronounced expansion of pro-inflammatory Mono and M1_Mac suggests a shift toward a stronger inflammatory phenotype (Fig. 4I). Further differential gene expression analysis demonstrated that, similar to T cells, more than twice as many genes were significantly downregulated than upregulated (Fig. 4J). These DEGs were mainly enriched in functional pathways related to leukocyte adhesion and proliferation of mononuclear cells (Fig. 4K). As shown in Fig. 4L, genes associated with M1 polarization (*S100a8*, *S100a9*, *Trem1*, *Il1b*, *Nlrp3*, etc.) and macrophage activation (*Itgam*, *Fcgr3*, *Rac2*, etc.) were significantly upregulated. In contrast, genes related to immune regulation and antigen presentation (*Cd74*, *H2-Ab1*, *Cd83*, *Pdcd1lg2*, etc.) were downregulated. Notably, a broad range of chemokines (*Cxcl2*, *Ccl3*, *Ccl4*, *Ccl2*, etc.) significant expression changes, suggesting dynamic modulation of immune cell recruitment.

Lastly, while the proportion of DCs was not significantly affected by DNCB treatment, their gene expression profiles exhibited substantial alterations (Figure S2A). These DEGs were primarily enriched in pathways related to leukocyte adhesion and migration (Figure S2B). Downregulation of antigen presentation and co-stimulatory genes, alongside upregulation of immune regulatory and inflammatory genes, suggests a transition of dendritic cells toward an inflammation-modulating state (Figure S2C).

### DNCB Treatment Induced Marked Transcriptional Alterations in the Endothelial Cell Population Within the Lesional Skin of AD Model Mice

**Fig. 5:**
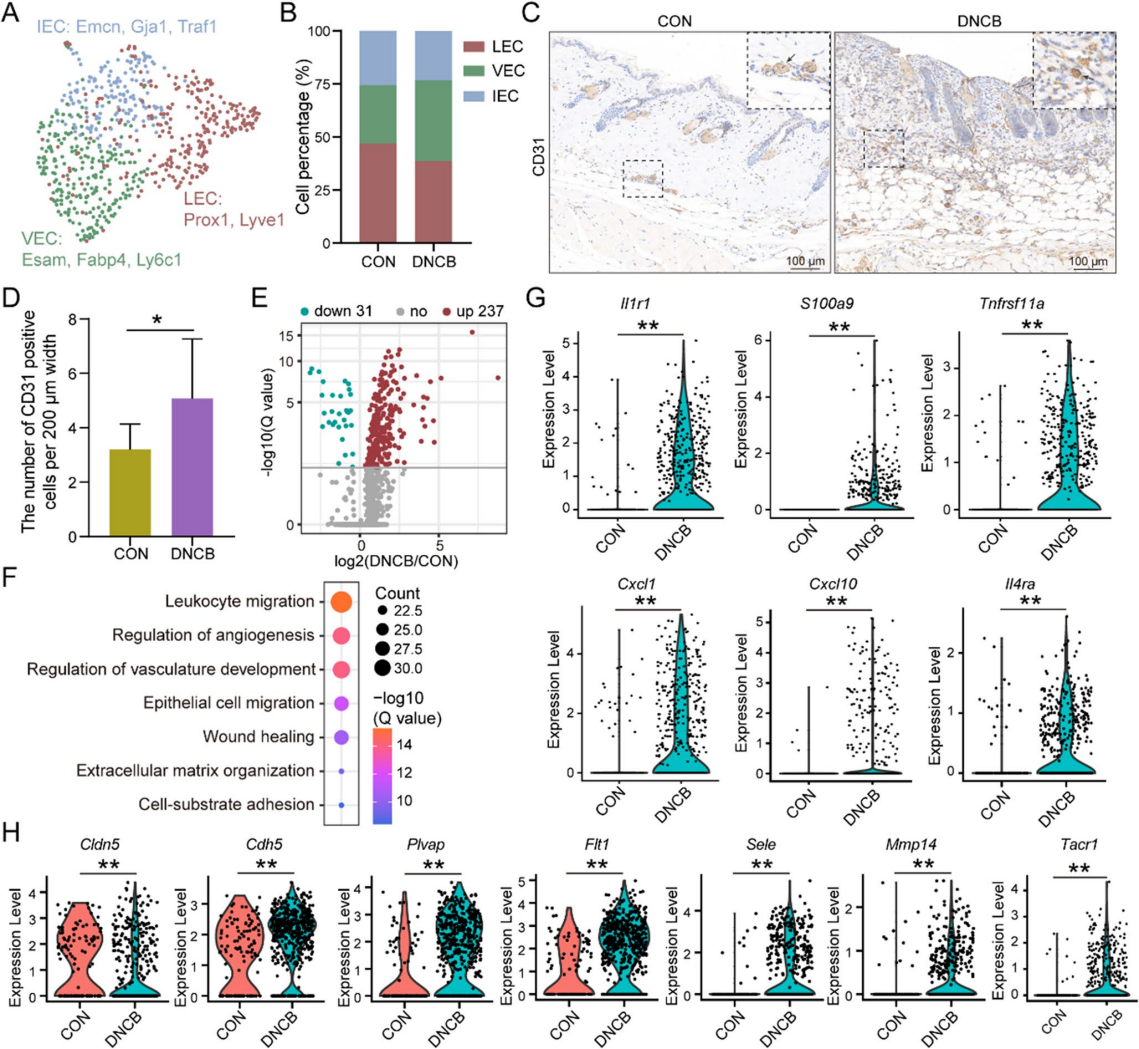
Endothelial cell transcriptional profiles are significantly altered in the DNCB-treated group. (**A**) Subpopulation of keratinocyte clusters colored based on three cell subtypes. (**B**) Cell percentages of three endothelial cell subtypes among two sample groups. (**C **and **D**) Representative IHC images and corresponding statistical bar graphs of CD31 between the control and DNCB-treated groups are shown. Scale bar, 200 μm. (**E**) The volcano plot shows the DEGs of endothelial cells between the control and DNCB-treated groups. (**F**) The bubble chart shows the GO enrichment results of DEGs in endothelial cells between the control and DNCB-treated groups. (**G**) The expression of inflammatory factors-related genes in control and DNCB-treated groups. (**H**) The expression of vascular remodeling-related genes in control and DNCB-treated groups. *: *P* < 0.05; **: *P* < 0.01

ECs in the skin were classified into three distinct subpopulations based on their transcriptional profiles: vascular endothelial cells (VECs), lymphatic endothelial cells (LECs), and inflammatory endothelial cells (IECs) (Fig. 5A). The distribution of the three endothelial cell subsets remained largely stable between the control and DNCB-treated groups (Fig. 5B). The overall increase in endothelial cell abundance following DNCB exposure likely reflects a global expansion of the EC compartment rather than changes in specific subtypes (Fig. 5C and D).

Further differential expression analysis revealed that DNCB exposure induced widespread transcriptional reprogramming in ECs, with enrichment of genes involved in “regulation of angiogenesis” and “vasculature development”, indicating vascular remodeling in response to inflammatory cues (Fig. 5E and F). Importantly, several genes related to inflammation and chemotaxis, including *Il1r1*, *S100a9*, *Tnfrsf11a*, *Il4ra*, *Cxcl1* and *Cxcl10*, were significantly upregulated in ECs following DNCB treatment (Fig. 5G). Moreover, the downregulation of *Cldn5* alongside the upregulation of *Cdh5*, *Plvap*, *Flt1*, *Sele*, *Mmp14*, and *Tacr1* in ECs upon DNCB treatment collectively indicates endothelial barrier disruption, inflammatory activation, and vascular remodeling within the lesional skin microenvironment (Fig. 5H).

### DNCB Induces Aberrant Fibroblast Subclusters Exhibiting Differentiation Arrest

**Fig. 6:**
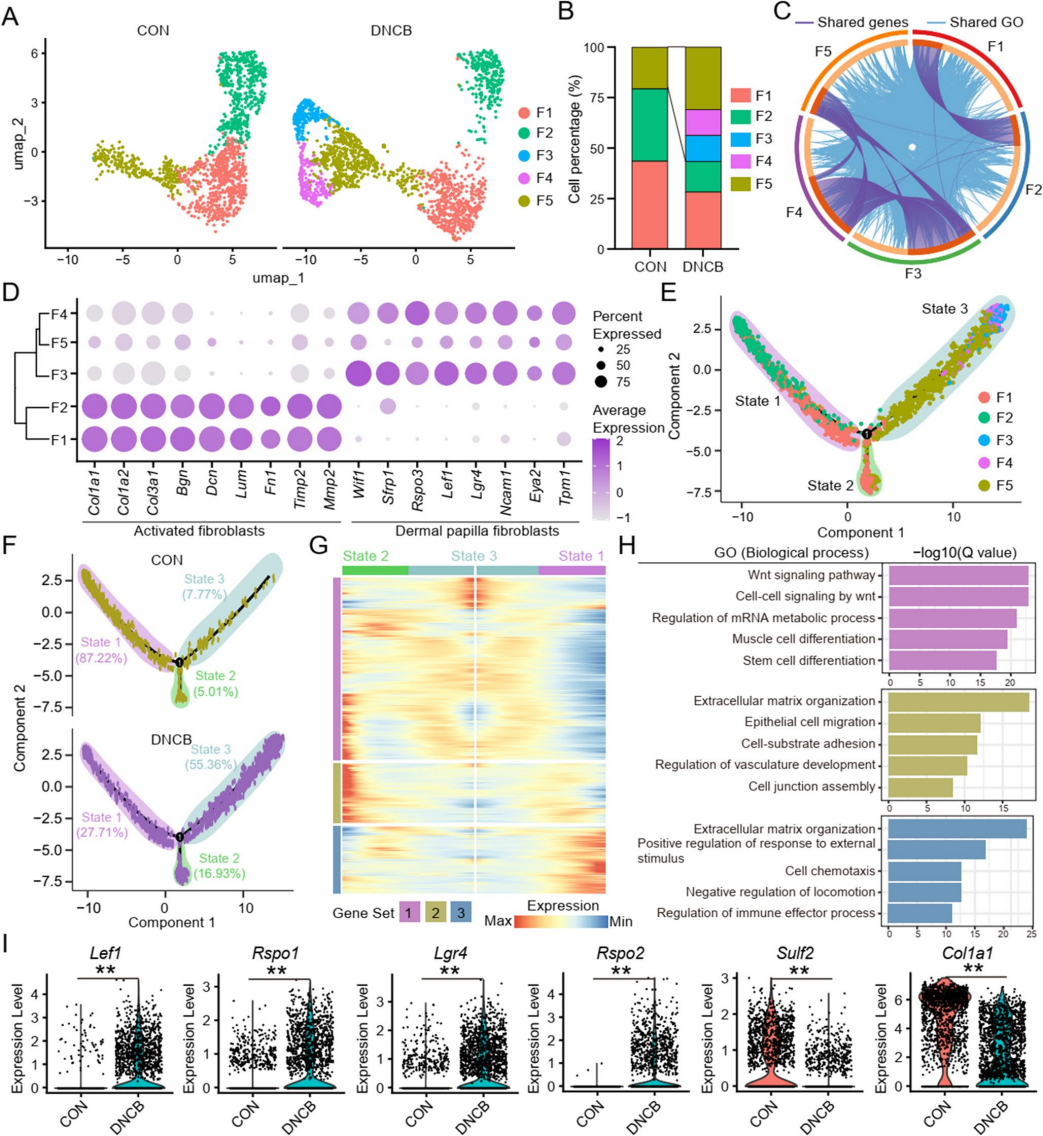
Fibroblasts cell fate are significantly altered in the DNCB-treated group. (**A**) Subpopulation of fibroblasts cluster colored based on five cell subtypes. (**B**) Cell percentages of five fibroblasts subtypes among two sample groups. (**C**) The chord diagram illustrates highly expressed genes shared across fibroblast subpopulations and their enriched GO pathways. (**D**) Bubble plot showing expression levels of subpopulation-associated genes. (**E**) Single-cell pseudotime developmental trajectory of fibroblast populations colored by three states, which were identified with two developmental fates. (**F**) Single-cell pseudotime developmental trajectory of fibroblast populations colored by two sample groups. (**G**) Pseudotime ordered heatmap of three DEG sets between two obvious fates at branch point one. (**H**) The graph shows the expression of the three DEG sets in the pseudotime trajectory. (**I**) The expression of DEG in control and DNCB-treated groups. **: *P* < 0.01

Fibroblast subpopulation analysis identified five distinct clusters (F1-F5) (Fig. 6A). Notably, two novel fibroblast subsets, F3 and F4, emerged specifically in the DNCB-induced group (Fig. 6B). These clusters shared transcriptional features with dermal papilla fibroblasts (F5), whereas F1 and F2 were more distinct and classified as activated fibroblasts (Fig. 6C and D). F3 was enriched for stemness-associated genes (e.g., *Notum*, *Rspo4*, *Wif1*), while F4 showed expression of neural-related genes (e.g., *Grid2*, *Nrg1*, *Sparcl1*) (Figure S3).

Subsequent pseudotime trajectory analysis of the entire fibroblast population revealed distinct dynamic transitions in response to DNCB exposure. Specifically, cell states 2 and 3 were markedly enriched in the DNCB group, and their gene expression profiles were predominantly associated with the Wnt signaling pathway, cell-substrate adhesion, and stem cell differentiation (Fig. 6E and H). Conversely, cells in state 1 were markedly decreased in proportion and were defined by gene expression signatures related to enhanced responses to external stimuli and suppressed locomotor activity (Fig. 6E and H). Further gene expression analysis revealed significant upregulation of key stemness-associated genes, including *Lef1*, *Rspo1*, *Rspo2*, and *Lgr4*, while activation-related genes such as *Sulf2* and *Col1a1* were markedly downregulated (Fig. 6I).

### Monocytes Emerge as Central Mediators of Ligand-Receptor Communication Following DNCB Treatment

**Fig. 7:**
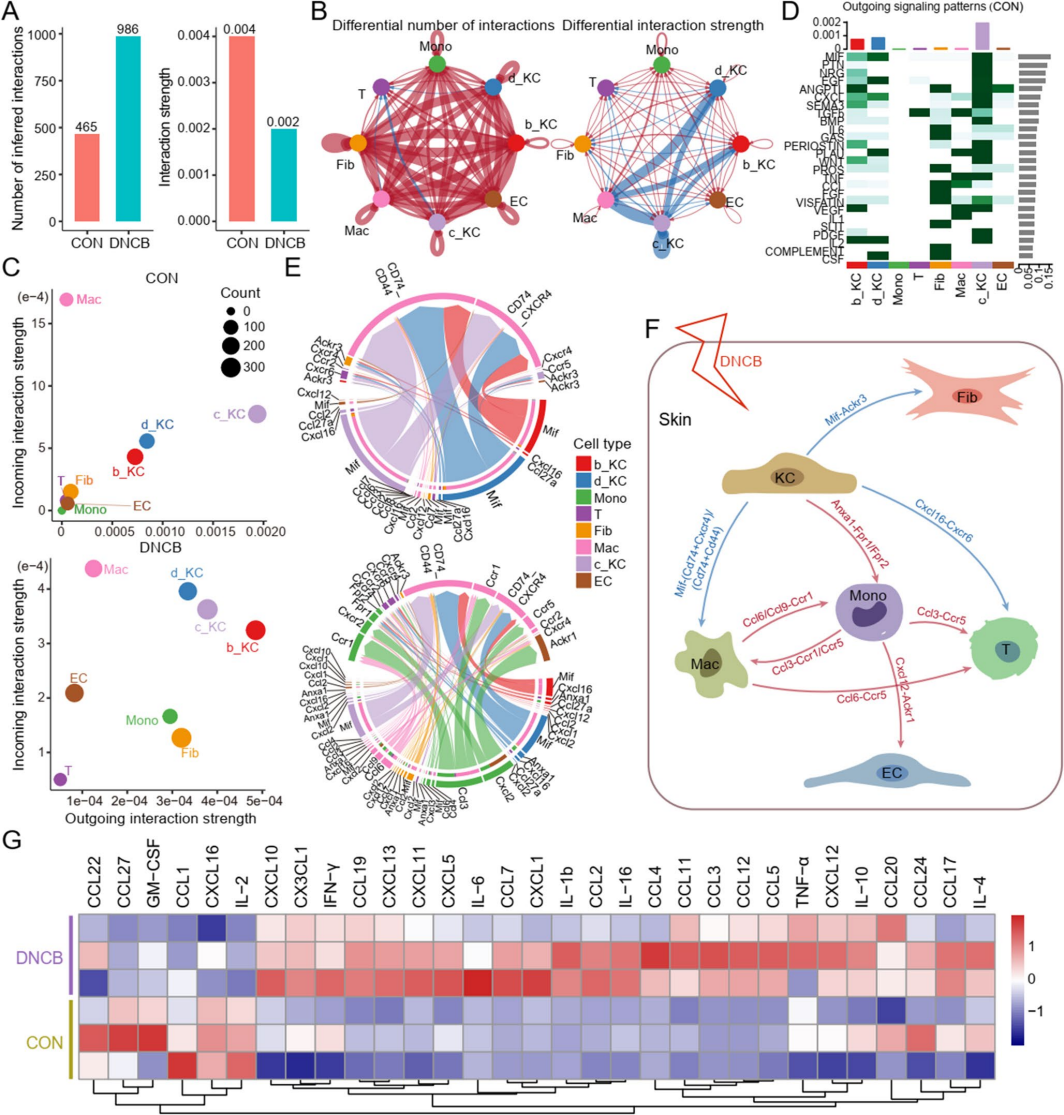
DNCB exposure fundamentally remodeled intercellular ligand-receptor signaling networks. (**A**) Bar chart showing interaction strength in skin of control and DNCB-treated groups. (**B**) Differential interaction number and differential interaction strength among the 8 main cell populations in control and DNCB-treated groups; red and blue indicate increase or decrease, respectively. (**C**) 2D spatial maps of the intensity of incoming interaction strength and outgoing interaction strength of 8 major cell groups in skin of control and DNCB-treated groups. (**D**) Heatmap of outgoing signaling patterns of 8 major cell populations in control skin. (**E**) Chord diagrams depict ligand-receptor interaction patterns among eight major cell populations in both control and DNCB-treated groups. **(F**) Schematic representation of DNCB-induced alterations in ligand-receptor signaling networks in atopic dermatitis. Red and blue denote increased and decreased interactions, respectively. (**G**) Heatmap showing the expression levels of multiple inflammatory cytokines and chemokines in control and DNCB-treated groups

We next investigated alterations in intercellular communication. Although the DNCB-treated group showed a greater number of cell-cell communication events compared to controls, the overall interaction intensity was diminished (Fig. 7A). This attenuation in signaling intensity was primarily observed between KC and Mac (Fig. 7B). Two-dimensional spatial communication mapping further revealed a marked enhancement of interaction strength among Mono in the DNCB group, whereas KC-mediated interactions were diminished relative to controls (Fig. 7C). Notably, in normal skin, KCs serve as the major source of outgoing signals; however, following DNCB exposure, this role shifted toward Mono (Fig. 7D and E). Due to the intrinsic complexity of cell-cell communication in heterogeneous skin tissue, we systematically examined the DNCB-induced alterations in incoming signals among various cell types and successfully delineated key regulatory networks underlying the inflammatory response (Fig. 7F, S4, and S5). Finally, multiplex cytokine assays demonstrated significant changes in inflammatory and chemotactic factors between the two groups (Fig. 7G). In particular, CCL3 (primarily secreted by Mono) was significantly upregulated, while CXCL16 (mainly derived from KC) was markedly downregulated. Collectively, these findings indicate that DNCB treatment reshapes the skin’s cellular communication landscape, redirecting the central signaling role from KC to Mono, thereby contributing to an inflammatory microenvironment.

## Discussion

AD is a complex chronic inflammatory skin disease marked by epidermal barrier dysfunction, immune dysregulation, and tissue remodeling [1, 29]. Although the DNCB-induced murine model has been widely used to study AD pathogenesis and screen therapeutic agents [8–10], the cellular and molecular mechanisms driving disease progression remain incompletely understood. In this study, the group size (*n* = 5) was selected with reference to previous DNCB-induced atopic dermatitis models reporting comparable sample sizes [30–32]. Furthermore, this design follows the 3R principles (Replacement, Reduction, Refinement), ensuring ethical animal use while preserving statistical validity. We used scRNA-seq to generate a comprehensive single-cell atlas of DNCB-induced lesional skin, uncovering major shifts in cellular composition, transcriptional states, and intercellular communication that closely parallel human AD pathology.

Consistent with human data [33–35], DNCB drives extensive immune cell infiltration, particularly of Th2/Th17-skewed T cells and inflammatory M1_Mac. Notably, T cells exhibited a transcriptional shift toward Th2/Th17 polarization, characterized by upregulated expression of *Il17a/f*, *Il23r*, *Stat3*, and *Jak2*/*Stat5* axis genes, which closely resembles the inflammatory signatures observed in lesional skin of AD patients [36–38]. The concomitant suppression of Th1 pathways exacerbates immune dysregulation, recapitulating pathogenic signatures observed in human AD across age groups and validating the translational utility of the DNCB model [39, 40]. Our results extend these insights by showing that this polarization is accompanied by a profound reconfiguration of the cutaneous cytokine network, with monocytes emerging as dominant communication hubs.

In AD, keratinocytes which form the structural backbone of the epidermis exhibit multifaceted dysfunction including increased proliferation, enhanced apoptosis, and impaired terminal differentiation [41–43]. Our pseudotime trajectory analysis further showed DNCB-induced skewing of keratinocyte fate toward immature, proliferative states at the expense of terminal differentiation, a pattern recapitulated in studies of lesional vs. non-lesional human skin [24, 41, 44]. Importantly, the disruption of epidermal barrier genes suggests that environmental allergens such as DNCB compromise the skin’s barrier functions in a manner consistent with filaggrin-deficient AD models [41, 45]. These findings implicate keratinocyte stress responses as both contributors to and targets of inflammation.

In addition to epithelial and immune changes, our analysis revealed significant reprogramming of endothelial and fibroblast populations. Endothelial cells in the lesional skin underwent transcriptional reprogramming indicative of vascular remodeling, increased permeability, and immune recruitment, as evidenced by *Il1r1*, *Cxcl1*, *Sele*, and *Plvap* upregulation. These changes parallel vascular alterations observed in human AD, including endothelial activation and increased angiogenesis [46–48]. Moreover, the emergence of DNCB-specific fibroblast subsets expressing stemness- and neural-associated genes indicates a degree of fibroblast plasticity under chronic inflammatory conditions. Although similar plasticity has been reported in fibrotic and chronically inflamed human skin [49, 50], the specific subsets observed in the murine AD model remain distinct. Previous single-cell studies have described novel fibroblast populations in human AD skin [23], yet the transcriptional profiles of the subsets identified here have not been reported, suggesting species- and context-specific fibroblast responses to chronic inflammation.

Perhaps most strikingly, cell-cell communication analysis uncovered a fundamental reorganization of intercellular signaling networks. In healthy skin, keratinocytes serve as central communication nodes, secreting cytokines that regulate immune and stromal cell behavior. However, in lesional skin, this role is overtaken by pro-inflammatory monocytes, which orchestrate pathogenic signaling via upregulated ligands such as CCL3, TNF, and IL-1β [51, 52]. The impact of this monocytes-derived signaling on recipient cells is profound and mediated by key upstream signaling hubs. The ligands TNF and IL-1β are potent activators of the NF-kB and MAPK pathways, while also influencing the JAK-STAT cascade [53]. This is corroborated by findings that T cells in lesional skin exhibit marked activation of NF-kB signaling (e.g., upregulation of *Nfkb1*, *Nfkbia*) and a skewing toward Th2/Th17 polarization, driven by the upregulation of critical genes including *Jak2*, *Stat3*, *Stat5a*, and *Stat5b*. Concurrently, the downregulation of TGF-β signaling molecules (*Tgfbr2*, *Smad2*, *Smad3*) disrupts homeostatic repression of inflammation [54]. Targeting these axes has clear translational appeal, as TNF blockade is well established in psoriasis and hidradenitis suppurativa [55], and IL-1 receptor antagonism with anakinra has shown efficacy in dermatologic and autoinflammatory disorders [56]. However, TNF inhibitors can paradoxically trigger eczematous or atopic phenotypes in a subset of patients [57], underscoring the importance of disease context and class-specific risks. Collectively, this signaling switch may represent a tipping point in chronic inflammation, and monocyte-derived cytokine signatures could serve as biomarkers to guide stratified therapy in refractory AD [58].

Despite the insights gained, several limitations must be acknowledged. First, while the DNCB model captures key hallmarks of human AD, the current single-cell approach lacks resolution to fully distinguish T cell subsets (e.g., Th1/Th2/Th17). Future studies employing immune cell enrichment followed by high-depth scRNA-seq could provide more detailed characterization of these critical populations [59]. Second, neutrophils and mast cells were not detected in our scRNA-seq dataset, possibly due to their fragility, low abundance, and susceptibility to tissue dissociation and enzymatic digestion [60, 61]. Third, the study focused on skin tissue and did not examine draining lymph nodes or systemic immune compartments that also contribute to AD pathogenesis. Lastly, while ligand-receptor analysis provides valuable inferences, functional validation of these signaling axes will be essential to confirm their pathogenic roles.

## Conclusions

In conclusion, our single-cell analysis provides a detailed overview of the cellular and transcriptional changes in DNCB-induced AD-like skin inflammation. The results highlight coordinated alterations across multiple cell populations, driven by changes in cell states and intercellular communication. This comprehensive single-cell characterization provides new insights into AD pathogenesis and identifies key cellular players, particularly monocytes and dysregulated keratinocytes, that may serve as potential targets for therapeutic intervention. The shared transcriptomic signatures between this murine model and human AD further support its translational relevance and underscore the value of scRNA-seq in decoding complex tissue pathology.

## Supplementary Information

Below is the link to the electronic supplementary material.

Supplementary Material 1 (DOCX 1.88 MB)

## Data Availability

The single-cell RNA-seq data generated from DNCB-induced AD mouse models in this study have been deposited in the Genome Sequence Archive (GSA, [https://ngdc.cncb.ac.cn/gsa](https://ngdc.cncb.ac.cn/gsa)) under the accession number CRA026856. The R code using for data processing is publicly available at GitHub at https://github.com/LWX-Lab/DNCB-data-code.git. Data supporting the findings of this study are available from the corresponding author upon reasonable request.
