# Supplementary material for: Single-Cell Transcriptomic Characterization of DNCB-Induced Mouse Model Reveals Atopic Dermatitis-Associated Skin Lesions in Skin Microenvironment
Source: Inflammation. 2026 Jan 24;49(1):63. doi: 10.1007/s10753-025-02391-5 (PMC12891011; doi:10.1007/s10753-025-02391-5)
Supplement: Supplementary file 1 — Supplementary Material 1 (DOCX 1.88 MB) [file 10753_2025_2391_MOESM1_ESM.docx]

**Supplementary figure and figure legends**


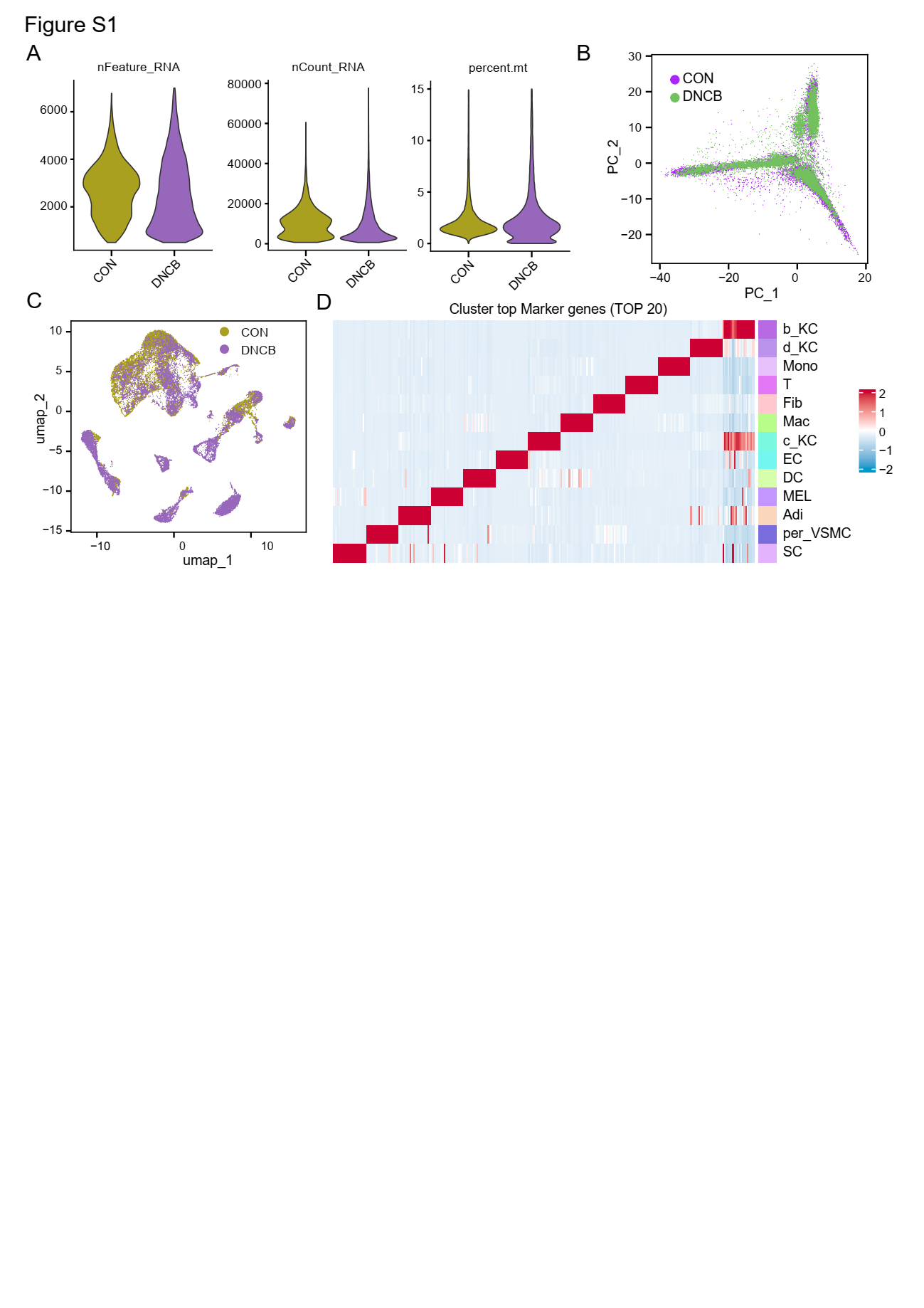


**Figure S1 Single cell sequencing information, quality control results**. (A) Violin plots demonstrate the number of genes (nFeature_RNA), unique molecular identifier (nCount_RNA), and percentage of mitochondria genes (percent.mt) in control and DNCB-treated groups. (B) PCA of each sample. (C) UMAP plot color-coded by cell source of control and DNCB-treated groups. (D) The heatmap showed the top 20 DEGs of each cell type.


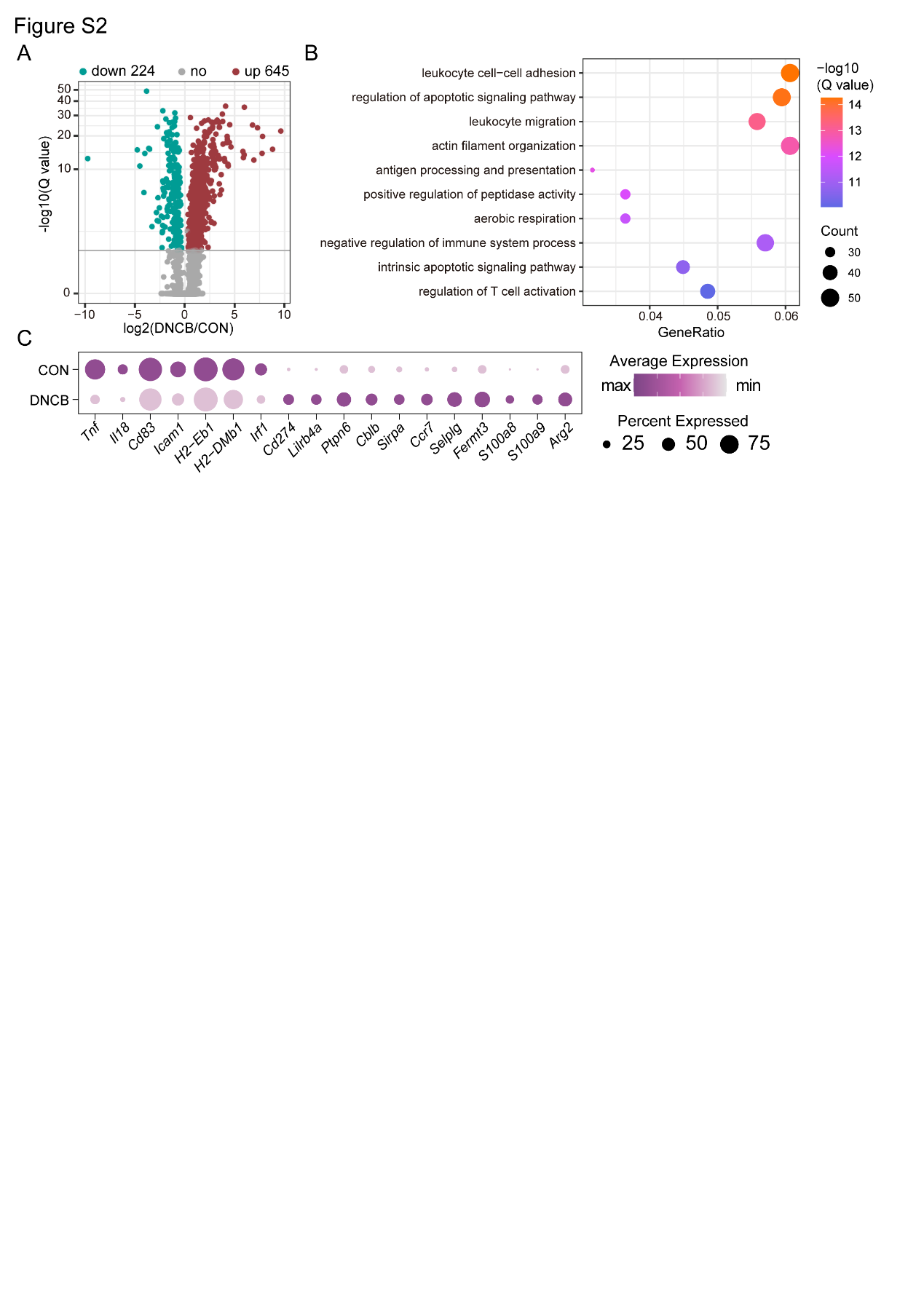


**Figure S2 Dendritic cell transcriptional profiles are significantly altered in the DNCB-treated group.** (A) The volcano plot shows the DEGs of dendritic cells between the control and DNCB-treated groups. (B) The bubble chart shows the GO enrichment results of DEGs in dendritic cells between the control and DNCB-treated groups. (C) Dot plot showing DEGs in dendritic cells from control and DNCB-treated groups.


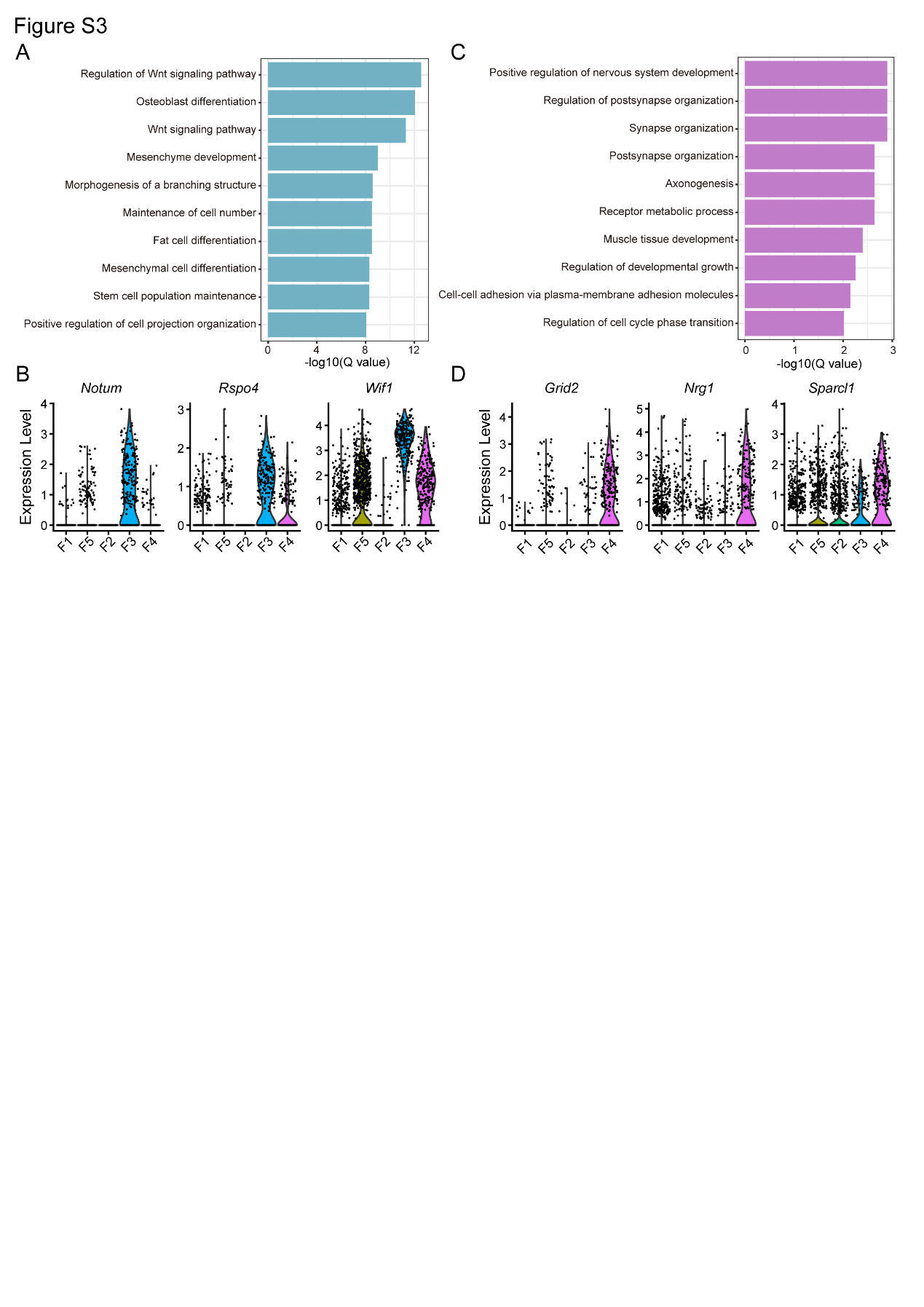


**Figure S3 DNCB treatment induced the emergence of two novel fibroblast subpopulations in cutaneous tissue.** (A) Bar plot of GO biological process enrichment analysis for signature genes in fibroblast subcluster F3. (B) Subcluster-specific expression of F3 fibroblast signature genes. Violin plots illustrate the distribution of three representative markers (*Notum*, *Rspo4*, *Wif1*) identified through differential expression analysis. (C) Bar plot of GO biological process enrichment analysis for signature genes in fibroblast subcluster F4. (D) Subcluster-specific expression of F4 fibroblast signature genes. Violin plots illustrate the distribution of three representative markers (*Grid2*, *Nrg1*, *Sparcl1*) identified through differential expression analysis.


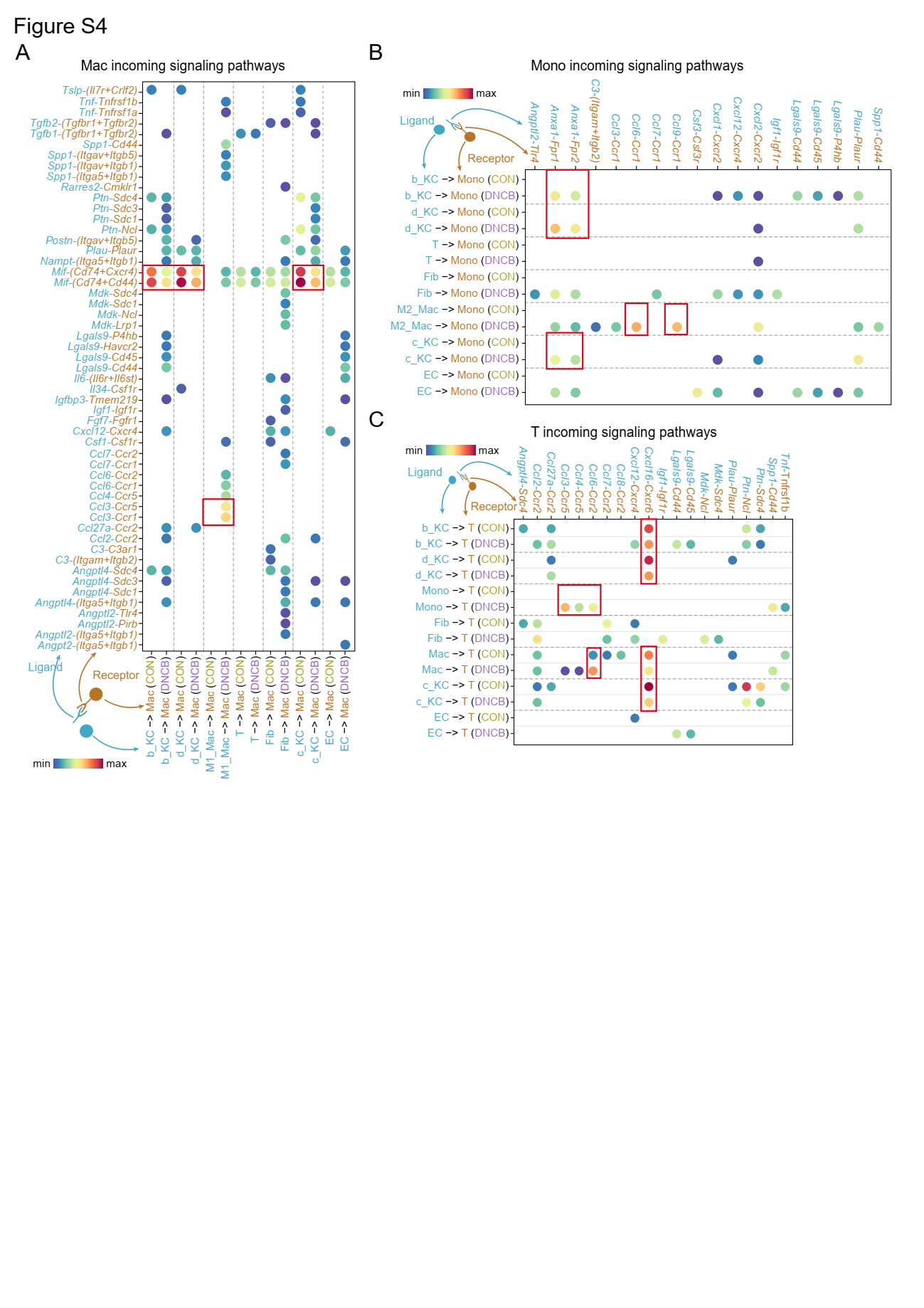


**Figure S4 DNCB-induced AD in mice demonstrated significant alterations in immune cell communication networks.** (A) The dot plot shows the expression levels of differentially regulated ligand-receptor pairs from other cell types to Mac in two groups. (B) The dot plot shows the expression levels of differentially regulated ligand-receptor pairs from other cell types to Mono in two groups. (C) The dot plot shows the expression levels of differentially regulated ligand-receptor pairs from other cell types to T in two groups.


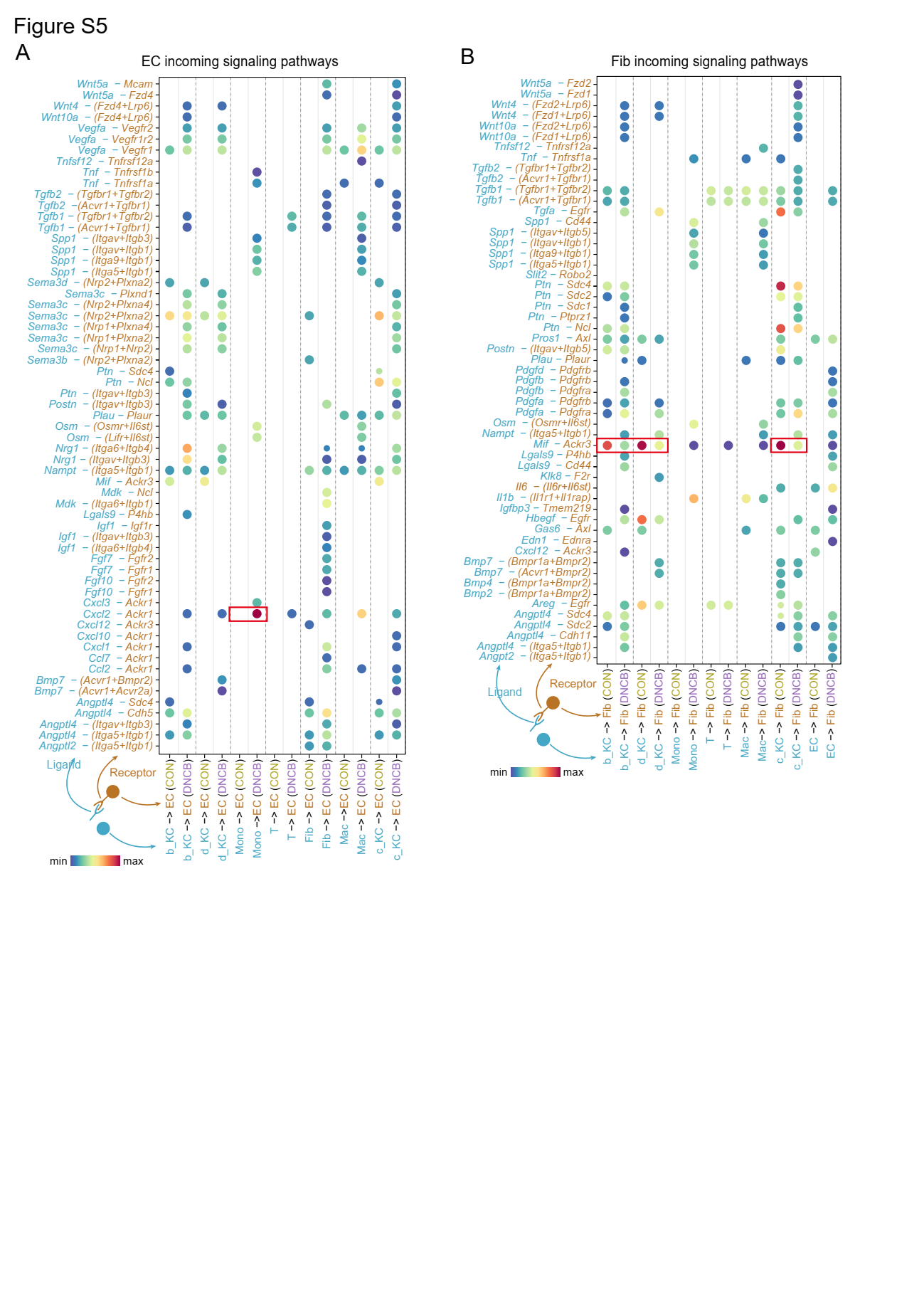


**Figure S5 DNCB-induced AD in mice demonstrated significant alterations in endothelial cell and fibroblasts communication networks.** (A) The dot plot shows the expression levels of differentially regulated ligand-receptor pairs from other cell types to endothelial cell in two groups. (B) The dot plot shows the expression levels of differentially regulated ligand-receptor pairs from other cell types to fibroblasts in two groups.
